# Outdoor spatial spraying against dengue: A false sense of security among inhabitants of Hermosillo, Mexico

**DOI:** 10.1371/journal.pntd.0005611

**Published:** 2017-05-17

**Authors:** Pablo A. Reyes-Castro, Lucía Castro-Luque, Rolando Díaz-Caravantes, Kathleen R. Walker, Mary H. Hayden, Kacey C. Ernst

**Affiliations:** 1 Center for Studies on Health and Society, El Colegio de Sonora, Hermosillo, Sonora, México; 2 University of Arizona, Tucson, Arizona, United States of America; 3 National Center for Atmospheric Research, Boulder, Colorado, United States of America; Centers for Disease Control and Prevention, UNITED STATES

## Abstract

**Background:**

Government-administered adulticiding is frequently conducted in response to dengue transmission worldwide. Anecdotal evidence suggests that spraying may create a “false sense of security” for residents. Our objective was to determine if there was an association between residents’ reporting outdoor spatial insecticide spraying as way to prevent dengue transmission and both their reported frequency of dengue prevention practices and household entomological indices in Hermosillo, Mexico.

**Methodology/Principal findings:**

A non-probabilistic survey of 400 households was conducted in August 2014. An oral questionnaire was administered to an adult resident and the outer premises of the home were inspected for water-holding containers and presence of *Ae*. *aegypti* larvae and pupae. Self-reported frequency of prevention practices were assessed among residents who reported outdoor spatial spraying as a strategy to prevent dengue (n = 93) and those who did not (n = 307). Mixed effects negative binomial regression was used to assess associations between resident’s reporting spraying as a means to prevent dengue and container indices. Mixed effects logistic regression was used to determine associations with presence/absence of larvae and pupae. Those reporting spatial spraying disposed of trash less frequently and spent less time indoors to avoid mosquitoes. They also used insecticides and larvicides more often and covered their water containers more frequently. Their backyards had more containers positive for *Ae*. *aegypti* (RR = 1.92) and there was a higher probability of finding one or more *Ae*. *aegypti* pupae (OR = 2.20). Survey respondents that reported spatial spraying prevented dengue were more likely to be older and were exposed to fewer media sources regarding prevention.

**Conclusions/Significance:**

The results suggest that the perception that outdoor spatial spraying prevents dengue is associated with lower adoption of prevention practices and higher entomological risk. This provides some support to the hypothesis that spraying may lead to a “false sense of security”. Further investigations to clarify this relationship should be conducted. Government campaigns should emphasize the difficulty in controlling *Ae*. *aegypti* mosquitoes and the need for both government and community action to minimize risk of dengue transmission.

## Author summary

Outdoor spatial spraying is commonly practiced by local governments as a prevention strategy for dengue and other *Aedes*-borne viruses. However, it has been publically criticized for its potential to reduce community-level actions such as clearance of mosquito habitat due to a resulting “false sense of security”. This study uses a paired social and entomological survey to analyze the association between residents’ perception that outdoor spraying prevents dengue and household level dengue fever prevention practices and entomological indicators in a city in northern Mexico. Our results suggest that those who view dengue is prevented by outdoor spatial spraying had 1) lower frequencies of elimination of potential mosquito breeding sites and higher frequencies of exposure to the vector, 2) higher entomological risk, and 3) a different socio-demographic profile than those who did not report it as effective. Our findings support the possibility that outdoor space spraying may lead to a “false sense of security” which reduces household engagement in prevention activities.

## Introduction

Dengue viruses (DENV), transmitted primarily by the *Aedes aeygpti* mosquito, are a serious and growing health threat around the world [1]. Each year, 390 million infections are estimated to occur, of which 96 million are symptomatic [2]. The recent development and implementation of the CYD-TDV vaccine is promising [3,4], but the principal strategy of dengue fever prevention programs remains the control of the *Ae*. *aegypti* mosquito population and prevention of vector-human contact [5–7].

Outdoor spatial spraying using ultra-low volume (ULV) fogging is one of the most common practices used by local governments to target adult mosquito vectors [8–10]. The insecticides (typically synthetic pyrethroids or organophosphates) are applied to outdoor areas using truck-mounted equipment or backpack sprayers [11,12]. The equipment is designed to disperse the insecticide in droplets between 5 and 25μ, small enough to create a fog that will drift throughout the treated area, killing adult mosquitoes [11,12]. Evidence for the efficacy of outdoor spatial spraying for dengue prevention and control is limited, and there is a strong need for more controlled studies [13]. Critics of outdoor space spraying for dengue control point out its inefficiency in penetrating poorly accessible spaces in and around homes as well as the use of chemicals that have no residual activity [8,14,15]. In addition, repeated use of particular insecticides has been associated with the development of resistance in the vector [16–20].

Outdoor space spraying can be performed proactively and reactively [9]. Currently, the World Health Organization (WHO) recommends limiting the use of spatial spraying to emergency situations in order to prevent an incipient epidemic or to halt one already in process [1]. Outdoor space spraying alone has not been proven effective in controlling outbreaks [21]. WHO recommends including outdoor space spraying in an integrated vector management plan that also involves the use of larvicides, the reduction of breeding sites, and personal protection measures to reduce human-vector contact [1].

This integrated approach demands a high level of community participation [22,23]. Recommended personal protection practices include the application of mosquito repellant and the use of window screens and doors to prevent mosquito entry into homes. Household and community vector control activities involve removal of trash that may collect water, periodic checking and dumping of standing water, and covering water storage containers. However, concern has been raised that outdoor space spraying could discourage the adoption of personal protection and vector control practices among some individuals due to what has been called a “false sense of security” [24–29]. From a political perspective, outdoor space spraying represents a visible action that transfers to the public a sense that governments are doing something to control mosquito-borne diseases. Could this lead to a diminished sense of risk and responsibility? Little is known about the social impact of outdoor space spraying on community responsiveness [28].

The objective of this study was to analyze the relationship between residents’ perception that outdoor space spraying prevents dengue and self-reported dengue fever prevention practices as well as the entomological risk among homes in the city of Hermosillo, Mexico. In addition, this study assessed the socio-demographic characteristics associated with positive attitudes towards outdoor space spraying.

## Methods

### Ethics statement

The study was approved by the University of Arizona Human Subjects Review Board.

### Study area

The current population in the city of Hermosillo is approximately 800,000 inhabitants and 194,000 households. This city is located in the Sonoran desert region of northwest Mexico (latitude 29°05’ N, longitude 110°57’ W) at an altitude of 216 meters above sea level [30]. The city has an average annual temperature around 25°C with daily maximum temperatures above 43°C from May to August and low annual levels of precipitation around 100 mm [31]. For the past three decades, dengue has been endemic in the city, causing several epidemics in recent years [32]. Local vector control efforts are coordinated through the Ministry of Health and use the guidelines of the National Center of Preventive Programs and Disease Control (CENAPRECE, Spanish acronym) [12]. In addition to public education and removal of containers that could serve as larval habitat, principal vector control activities are applications of insecticides to kill either the larvae or adult mosquitoes. Insecticides targeting adult mosquitoes are applied using ULV fogging, usually from truck-mounted equipment and indoors using backpack sprayers.

Outdoor space spraying is carried out only in defined spraying priority areas. Priorities for spraying are set by epidemiologic (number of probable cases) and/or entomologic (% of positive ovitraps, mean of eggs by block) indicators. If a probable case is identified outdoor space spraying is conducted in at least nine blocks around the household of a probable case who had symptom onset within the past 10 days. Even if additional cases are identified in the area, spraying will not be conducted in the same area more than once in every 15 days [12]. Indoor spraying is limited to houses in the immediate vicinity of a confirmed or suspected human case of dengue or other arbovirus. During the time of this study, the insecticide used for outdoor space spraying was the organophosphate chlorpyrifos, due to reports of pyrethroid resistance in the vector population.

### Study design

**Fig 1:**
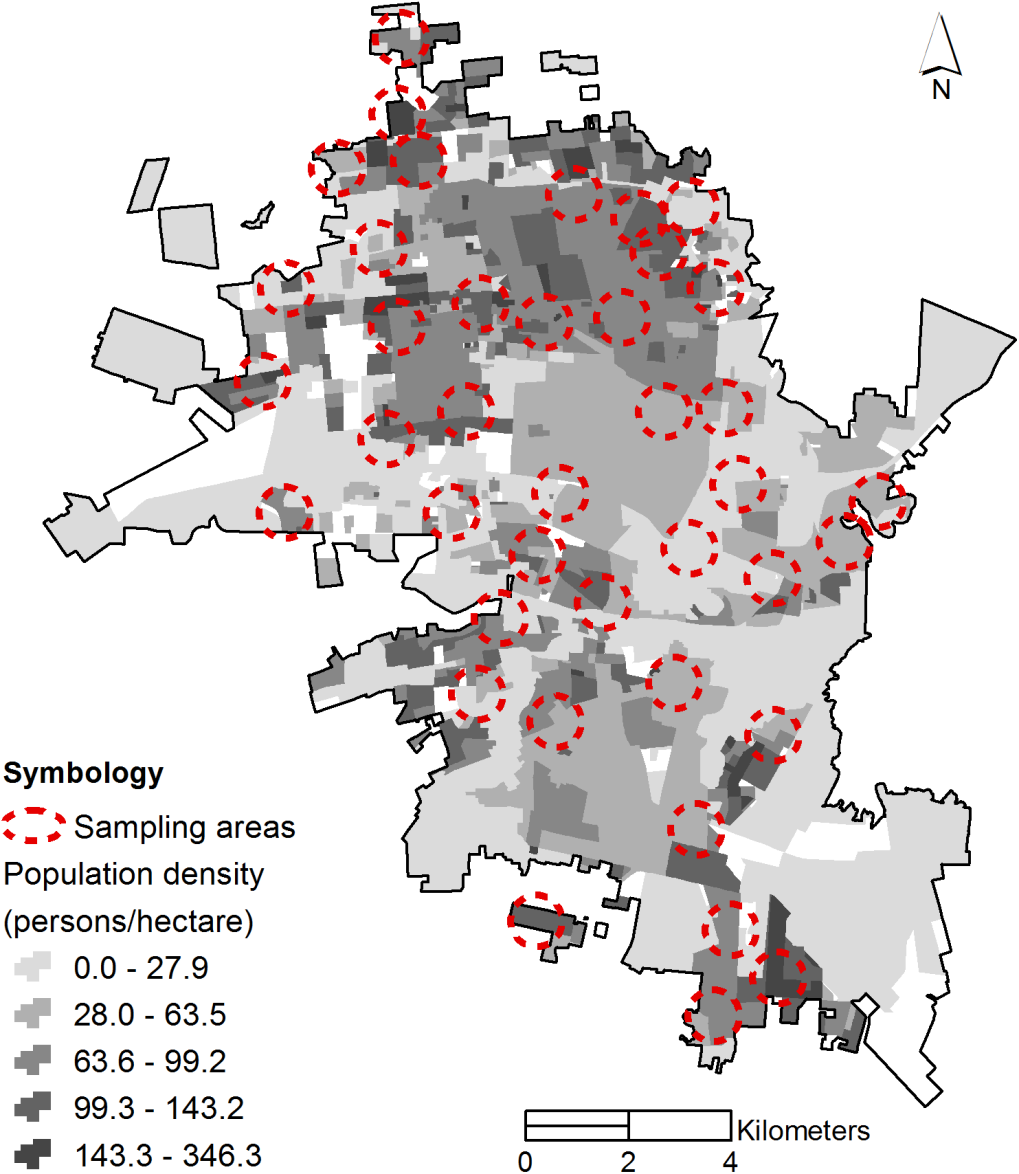
Sampling areas distribution.

A cross-sectional study was conducted from August 12 to September 3, 2014 as part of a broader cross-border research project (Mexico-USA) examining the potential for further dengue emergence in the region. As part of the broader study, 40 sites had been selected in Hermosillo that were at least 1km apart for placement of traps for adult *Ae*. *aegypti*. Ten households were selected around each of the 40 trapping sites in the following manner: recruitment began in the household across the street from the residence where the adult trap was placed, every third house was approached for recruitment in the block around the trap site and the streets one street away from the central block. This strategy enabled good geographic coverage for the survey (Fig 1). Households were included when one of the residents over age 18 was available and willing to participate after giving oral consent. Recruitment was conducted from 8:00 am to 8:00 pm to accommodate different householder schedules. If no one was present during an initial attempt households were revisited one time on a different day, including weekends, and at a different time to attempt recruitment. If the resident was not available on the second attempt, the household was replaced by going to the houses adjacent until a participant was identified. Potential replacement households were only visited once. A total of 1251 households were visited. Of these, 171 actively refused (13.7% refusal rate), and no one was at home in another 688 households, yielding an overall response rate of 31.8%.

### Information sources and collection

The survey included a social science component and an entomological component. For the collection of socio-demographic information and that of prevention practices, an adaptation of the Knowledge, Attitudes and Practices questionnaire prepared by Ernst et al. [33] was used. The survey tool was previously applied in the cities of Key West, Florida and Tucson, Arizona, USA in 2012. Four teams of two people each were organized. While one person orally administered the questionnaire, the other member of the team inspected the front and back yard looking for potential mosquito oviposition sites. All field investigators were trained over a period of one week on the survey and entomological collections with most having a background in biology. Any object that could contain water was recorded and assessed for the presence of water and immature mosquitoes. If immature mosquitoes were present, a sample of larvae and all pupae were collected and brought to the University of Arizona for speciation.

To define the study groups, respondents’ were asked *“In your opinion*, *how is dengue fever prevented*?”. This was an open-ended question with pre-coded responses based on a priori expectations. As respondents mentioned unsolicited responses that matched the pre-coded responses surveyors marked them as yes. If there was no pre-coded response, “other” was marked and the response was noted. Pre-coded responses included: I do not know, empty water containers, discard water containers in backyards (descacharrización), application of residual insecticide/larvicide by the Health Ministry, spraying insecticide from trucks on street, and/or some other. Those respondents who reported “spraying insecticide from trucks on street” were considered to perceive that outdoor space spraying prevented dengue fever (OSS = 93) while those who did not include outdoor spraying were not considered to perceive outdoor space spraying as preventing dengue (NOSS = 307). The variables potentially associated with perception of outdoor space spraying as preventive are summarized in Table 1.

**Table 1:** Description of study variables.

Category	Variable	Description
OSS perception	OSS/NOSS	Perception of OSS as a dengue prevention strategy (binary/categorical)
Characteristics of respondent	Gender	Gender of respondent (binary/categorical)
	Age	Age of respondent (continuous)
	Basic schooling	≥ 9 years of school (binary/categorical)
	Household Index of Goods (HIG)	The *HIG* was created based on nine goods (radio, TV, refrigerator, laundry machine, car, computer, landline telephone, cell phone, and Internet). Values were normalized at a 0 to 1 scale with the formula: *HIG = (X–X*_*min*_*)/(X*_*max*_*−X*_*min*_*)* (continuous)
	Dengue history	Have had one or more dengue cases self-reported as laboratory confirmed in the household (binary/categorical)
	Media sources for dengue information	Number of media sources from which one received information about dengue prevention during the last two months (counts)
Prevention practices within the household	Frequency of 17 prevention practices	Frequency of 17 prevention practices were estimated through five-point Likert items ranging from 1 = never to 5 = always (ordinal)
Entomologic risk	Total containers per household	Number of total containers in the backyard (counts)
	Wet containers per household	Number of wet containers in the backyard (counts)
	Positive containers per household	Number of positive containers to immature *Ae*. *aegypti* mosquitoes in the backyard (larvae and/or pupae) (counts)
	Larvae presence/absence	Positive houses to *Ae*. *aegypti* larvae (binary/categorical)
	Pupae presence/absence	Positive houses to *Ae*. *aegypti* pupae (binary/categorical)

### Statistical analysis

To determine whether there were significant differences between the socio-demographic characteristics of the OSS and NOSS groups, Chi^2^ and Mann-Whitney U tests were used for categorical and continuous variables, respectively.

#### Frequency of practices

Median and Interquartile Range (IQR) were estimated for the list of practices of prevention in the houses and significant differences between the groups were determined using the Mann-Whitney U test.

#### Entomological risk

Container counts were stratified by OSS and NOSS groups and comparisons were made for number of containers, number of water-filled containers, and number of containers with immature mosquitoes. Mixed effects negative binomial regression for clustered data was used to calculate the Rate Ratio (RR) to estimate the association of the OSS perception with the density of total of containers, containers with water, and containers positive for immature *Ae*. *aegypti* mosquitoes while Odds Ratios (OR) were calculated using mixed effects logistic regression to determine the association with presence/absence of larvae and pupae in the houses.

Differences and associations were considered statistically significant at p<0.05; and 95% confidence intervals (C.I.) were estimated for OR and RR. ArcGIS 10.1 was used for map preparation and STATA 13 was used for all statistical analyses.

## Results

### Descriptive analysis and profile of OSS respondents

Residents of 400 houses were surveyed; the OSS group constituted 23.3% of the sample. Most individuals knew that the government used insecticides to control mosquitoes. When asked, “What methods does the government use in Hermosillo to control mosquitoes?” 81.7% of OSS reporters cited the use of insecticides with less (69.7%) in the NOSS group, p = 0.05). There were no other significant differences in reported government-led activities between the two groups. (Table 2).

**Table 2:** Residents profile by OSS perception.

Factors		OSS (n = 93)	NOSS (n = 307)	p-value ^a^
**Continuous variables**		**Median (IQR)**	**Median (IQR)**	
Age		50 (40, 59)	42 (30, 54)	<0.001
HIG		0.857 (0.571, 1.000)	0.857 (0.571, 1.000)	0.498
Number of media sources of dengue information within the last two months		1 (0, 1)	1 (0, 2)	<0.05
**Categorical variables**		**n (%)**	**n (%)**	
Gender	Male	30/93 (32.3)	83/304 (27.3)	0.354
	Female	63/93 (67.7)	221/304 (72.7)	
Basic schooling	Yes	81/93 (87.1)	264/306 (86.3)	0.839
	No	12/93 (12.9)	42/306 (13.7)	
Dengue history	Yes	9/89 (10.1)	22/302 (7.3)	0.386
	No	80/89 (89.9)	280/302 (92.7)	
Knowledge about local government vector control activities	Surveillance	1/93 (1.1)	6/307 (1.9)	0.571
	Draining stagnant water	9/93 (9.7)	41/307 (13.4)	0.347
	Distribution of information	12/93 (12.9)	51/307 (16.6)	0.390
	Spraying	76/93 (81.7)	207/307 (69.7)	<0.05
	Use of larvicides	42/93 (45.2)	133/307 (43.3)	0.754
	Other	10/93 (10.8)	36/307 (11.7)	0.797

a. Chi2 and Mann-Whitney U tests for categorical and continuous variables, respectively

The OSS group was generally older (median age 50 years) than the NOSS group (median age 42 years) (p<0.001). Gender was relatively equivalent between the groups (OSS = 67.3%; NOSS = 72.7%) with the majority of respondents being women. There were no differences in terms of basic schooling (both around 87%) or in the household index of durable goods (HIG). A higher proportion of OSS than NOSS respondents reported dengue cases in the home (15.7% versus 11.6%) but this difference was not significant (Table 2).

The OSS and NOSS groups also showed significant differences in the number of media sources from which they had received information about dengue within the last two months (p<0.05). While about half of all respondents received information about DF prevention through television, the OSS group typically received information from fewer sources than the NOSS group.

### Frequency of prevention practices: OSS vs. NOSS

**Fig 2:**
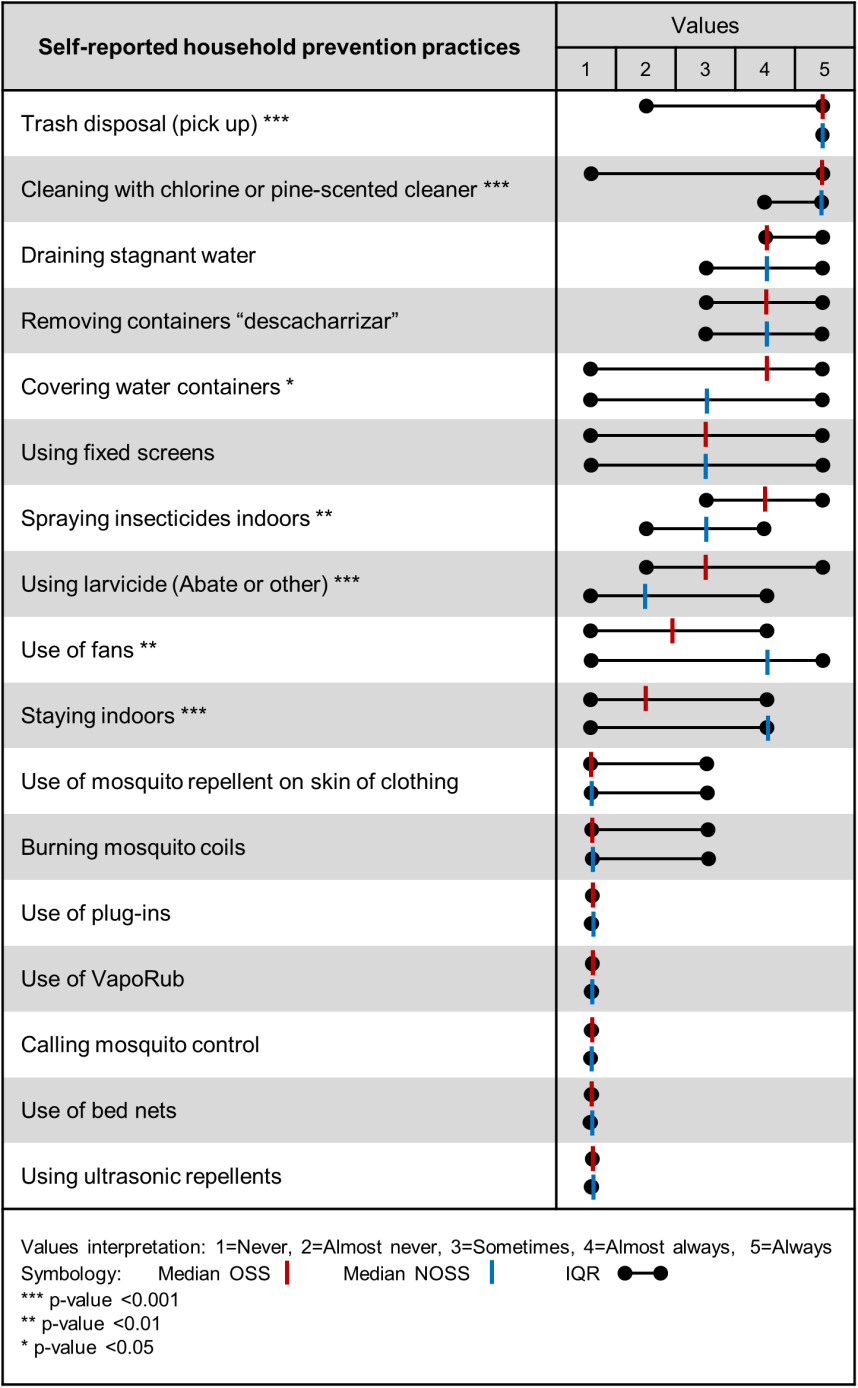
Comparison between frequencies of household dengue prevention practices by outdoor spatial spraying perception.

Overall, the most frequently performed practices (4 = almost always and 5 = always) included trash disposal, cleaning with chlorine and pine-scented cleaner, removing stagnant water, disposing of containers (*descacharrizar*), and covering water containers. Occasional practices (3 = sometimes) included use of mosquito nets, spraying inside of the house, using fans, staying indoors, and using larvicides such as Abate. Other practices, including the use of repellants, were rarely performed. Fig 2 shows the frequencies of dengue prevention practices for all 400 homes subdivided into the two groups (OSS and NOSS). The OSS groups showed lower frequencies in cleaning practices using bleach (OSS: 5 [1,5] vs. NOSS: 5 [4,5], p<0.001), using fans (OSS: 2.5 [1,4] vs. NOSS: 4 [1,5], p<0.01), and staying indoors (OSS: 2 [1,4] vs. NOSS: 4 [1,4], p<0.001). Although the medians regarding the frequency of trash disposal were the same for both groups, the first quartile of the OSS respondents reported a much lower frequency of trash disposal (almost never) than the first quartile of NOSS respondents (almost always) (OSS: 5[2,5] versus NOSS 5 [4,5], p<0.001). Conversely, the OSS group exhibited a higher frequency for spraying insecticides inside the house (OSS: 4 [3,5] vs. NOSS: 3 [2,4], p<0.01) and application of larvicides (OSS: 3 [2,5] vs. NOSS: 2 [1,4], p<0.001). Likewise, they reported covering water containers more frequently (OSS: 4 [1,5] vs. NOSS: 3 [1,5], p<0.05).

### Entomological risk

In the outdoor premises of OSS homes, a total of 1396 containers were identified (15.0 per household), of which 26.3% contained water vs. 3632 containers in the NOSS group (11.8 per household) with 30.1% containing water in the NOSS group. Within the OSS group, 11.4% of the containers were positive for larvae/pupae vs. 6.3% of water-holding containers in the NOSS group. All the entomological indices were higher of the OSS group; 23.7% of their houses were infested with larvae vs. 16.9% in the NOSS group, 18.3% were infested with pupae vs 9.1% in the NOSS group, 11.4% of the water-holding containers were infested with immature mosquitoes vs. 1.9% in the NOSS group, and there were 4.5 positive containers per 10 houses inspected vs. 2.2 per 10 in the NOSS group (Table 3).

**Table 3:** Entomological counts by outdoor spatial spraying perception.

Entomological counts	OSS (n = 93)	NOSS (n = 307)	Total (n = 400)	p-value ^a^
Total containers	1396 (15.0 per HH)	3632 (11.8 per HH)	5028	<0.05
Wet containers	367 (3.9 per HH)	1093 (3.5 per HH)	1460	0.39
Containers with immature *Ae*. *aegypti*	42 (4.5 per 10 HH)	69 (2.2 per 10 HH)	111	<0.01
Positive household (larvae)	22 (23.7%)	52 (16.9%)	74	0.144
Positive household (pupae)	17 (18.3%)	28 (9.1%)	45	<0.05

a. p-values based on Chi2 test and negative binomial distribution for proportion and count data, respectively

Mixed effects negative binomial regression analysis showed the number of containers positive to immature *Ae*. *aegypti* was 92% higher in the OSS group compared to the NOSS group (RR = 1.92; 95% C.I.: 1.15, 3.21), but there was no significant difference in the number of total containers and containers with water. The OSS group also had significantly more containers with *Ae*. *aegypti* larvae or pupae (RR = 2.01; 95% C.I.: 1.20, 3.36) (Table 4).

**Table 4:** Association between entomologic risk in backyards and outdoor spatial spraying perception.

Outcome variables	Measure of association^a^
**Containers**	**RR (95% C.I.)**
Total containers	1.10 (0.90, 1.35)
Wet containers	0.95 (0.76, 1.20)
Positive containers to immature *Ae*. *aegypti*	1.92 (1.15, 3.21)
**House positivity (presence/ absence) to immature *Ae*. *Aegypti***	**OR (95% C.I.)**
Positive houses for *Ae*. *aegypti* larvae	1.59 (0.84, 3.00)
Positive houses for *Ae*. *aegypti* pupae	2.20 (1.08, 4.48)

a. OSS vs. NOSS (reference group). RR>1: higher rate of containers in OSS group. OR>1: higher odds of house positivity for the OSS group. Significant associations were considered when the 95% C.I. did not include 1.00

Likewise, the mixed effects logistic regression analysis indicated the OSS group were two times more likely to have the presence of pupae (OR = 2.20; 95% C.I.: 1.08, 4.48). However, there was no significant association with presence of larvae (Table 4).

## Discussion

The results indicate differences in the frequency of dengue prevention practices and entomological risk associated with respondents’ view of outdoor space spraying, supporting the argument of a “false sense of security” associated with outdoor space spraying. Respondents who reported outdoor space spraying (the OSS group) as a dengue prevention strategy exhibited lower frequencies of cleaning and trash disposal, greater use of chemical pest controls around the house and had a higher number of positive containers with *Ae*. *aegypti* larvae/pupae around their houses. Overall, these findings suggest that residents who cite outdoor space spraying as a way to prevent dengue fever may be less likely to remove potential breading sites that may provide immature *Ae*. *aegypti* habitat and subsequently may produce more vectors in their yards than residents who do report that outdoor space spraying prevents dengue.

**Fig 3:**
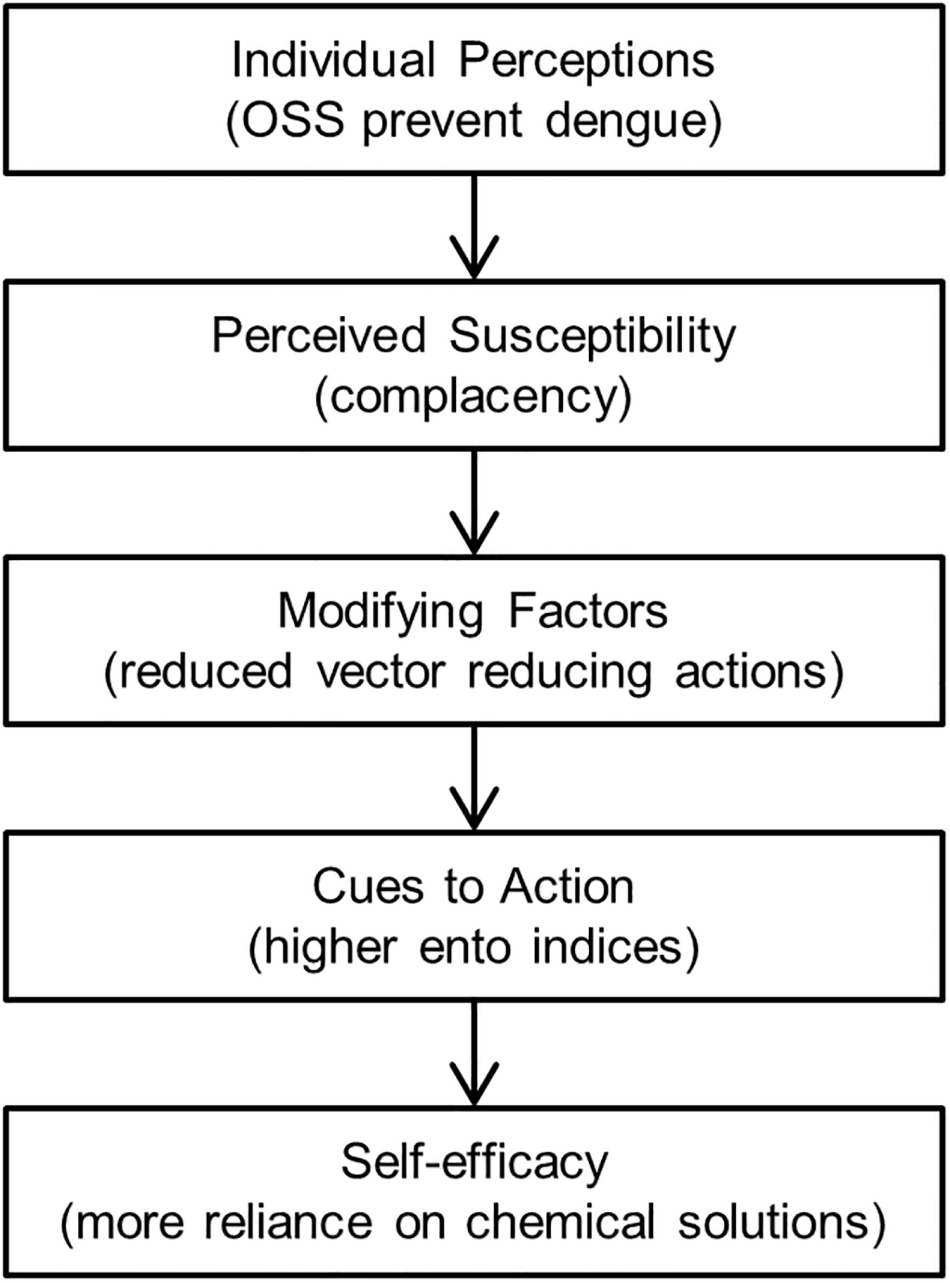
Pathway of associations between OSS perception, residents’ prevention practices and entomological indicators.

These associations may be best explained through social cognitive theory, specifically the health belief model [34]. The health belief model is a well-established theoretical framework for understanding the influences on the uptake of health behaviors. There are two primary components of the model that may be on the pathway of the association between awareness of ULV and uptake of specific prevention strategies. Individual perceptions, including the perceived disease risk, may be reduced with awareness that the government is initiating control measures in the community. If these government efforts are perceived as effective then this may reduce the perceived benefits of taking individual actions to further reduce disease risk. We propose the following pathway may lead to the associations identified in this analysis (Fig 3).

This pathway is supported by the negative association between OSS and frequency of trash disposal and the positive association with entomological indicators. Although there was no difference in the probability of larvae presence, the higher probability of finding pupae among the OSS group could be related to the lower frequency of trash disposal, providing more time for the mosquito to reach its pupal stage. This finding is consistent with results reported by Espinoza et al.[27], who found a higher number of positive containers in houses where outdoor space spraying took place in comparison to those where an education campaign was implemented. The authors even noted a reduction in the efficiency of educational intervention when combined with spraying, associating this fact with the possible effect of a “false sense of security” within the community; however, they did not conduct any surveys to directly assess the community knowledge of, or attitudes towards, spraying.

In our study, the OSS group did, however, report covering water containers with higher frequency than the NOSS group, a practice that can reduce the risk of transmission when this is accompanied by other community environmental management activities. Further information would have to be collected to understand whether this practice was undertaken explicitly to reduce mosquito populations.

Additionally, people in favor of OSS reported a higher frequency of the use of insecticides and larvicides inside and around their homes. Citing OSS as a prevention strategy may be part of a wider acceptance of the use of insecticides as a method of vector control.

Studies focused on the use of larvicides such as temephos (Abate, commonly used in Hermosillo) have consistently shown a reduction of entomological indexes at the community level [35–37]. The combined use of this practice with other chemical control methods has not, however, shown a sustained reduction in larval indices, and the links to actual reductions in dengue transmission are tenuous [37]. Some authors have attributed these sub-optimal results to the potential “false sense security” associated with the use of temephos; residents simply leave containers to be treated rather than removing them [38,39]. In our study, we were asking specifically about practices of the household respondent and not the government treatment of containers with larvicide. This may mean that larvicide is used in lieu of actual removal of containers. If the larvicide being used is not applied frequently enough, or if the *Ae*. *aegypti* populations have become resistant to the larvicide, this may result in higher container counts. The OSS group reported lower frequency in practices of trash disposal, as well as higher density of mosquito-positive containers. This suggests that a greater reliance and trust in chemical solutions as compared to physical solutions, such as removal of containers, may be present in the OSS group compared to the NOSS group. The lower engagement in environmental management by this group may impact the establishment of new breeding sites and the efficiency and intensity required for effective larvicide use.

Householders aware of OSS were more likely to use household insecticide sprays to prevent mosquitoes. This may be associated with the higher densities of mosquitoes that are identified in their surroundings and is more likely a result of the higher entomological indices than in the causal pathway of the association between OSS and entomological indices.

Ideally measures taken to reduce the vector density are coupled with practices to reduce contact with the vector. A key recommendation is avoidance of peak biting hours. Residents responding that OSS prevents dengue almost never use fans or stay indoors to avoid mosquito bites. This may relate to a perception of lower risk of mosquitoes in the environment following spray events. Ensuring that messaging includes information about the limitations of spraying is essential. Outdoor space spraying from trucks has a limited penetration into homes, reducing its efficiency indoors [15]. Additionally, the chemical used by the government for OSS does not have significant residual activity [8,14,15]. Spraying also does not guarantee constant protection from mosquito bites; so avoiding exposure to mosquitoes at periods of higher mosquito activity, may also reduce human/vector contact and DF transmission risk [40]. This is a particularly relevant issue in the arid environment of the city of study, especially during the months with a peak incidence of DF [41]. The high temperatures during the day can shift human activity outdoors to dawn and dusk when temperatures are lower, but the vector is more active.

The association of OSS perception with older populations may be related to historical memory. Between 1940 and 1970, vector control campaigns were heavily centralized throughout Latin America with vertically-delivered programs that did not directly involve communities. This may contribute to the perception in older community members that only government officials are equipped with the necessary training and resources to carry out control activities, reducing their active engagement [42]. Indeed there is an average eight year difference between the groups which may relate to changing norms in public perspective. Younger generations may no longer perceive that outdoor space spraying is the best strategy for DF prevention and control strategy. Campaigns that target older age groups to motivate action may be important to consider for future public health messaging and community participation in DF prevention and control [43].

Our study was based on whether individuals reported that OSS prevented dengue. There was no information available about local spraying activities. We did not have access to entomological and/or epidemiological surveillance information, however, the official guidelines indicate that OSS actions are based on results of vector and epidemiological surveillance as outlined previously. Local spraying activities and geographic variability in vector and disease distribution could influence individuals citing OSS as a way to prevent dengue. However, awareness of government spraying was high in both groups. The higher entomological indices may be a result of other factors in the environment that provide more habitat for the *Ae*. *aegypti* mosquitoes. Hermosillo, however, is relatively flat so there is no altitudinal gradient that would influence the densities. Further, there was at least one OSS respondent in 36 of the 40 clusters sampled, and the mode number of OSS respondents was 2 within a cluster indicating that the explanatory variable (OSS) was not entirely localized. Associations remained even when controlling for cluster within the mixed effects analysis. Future studies benefited from including surveillance data and local government prevention activities to provide context to this analysis. Unlike many regions of Mexico with dengue transmission, Hermosillo is an arid city which may mean results are not completely generalizable to other areas. However, Hermosillo follows the standard dengue prevention activities set by national guidelines which suggest these findings should be compared with results from other localities in Mexico.

This study is limited by the design. Cross-sectional studies preclude the ability to establish if exposures of interest precede the outcome; therefore, there is no directionality in our associations, and the outcomes observed could precede the outcome. In addition, the frequency questions for prevention practices were not designed to obtain exact numbers of times when each practice is performed and all frequencies were self-reported. Checking backyards made it possible to corroborate reported frequencies of refuse collection and resulting entomological indices; however, it was not possible to corroborate the other prevention practices or their impact on entomological indices and dengue incidence. Although the survey was not probabilistic, the geographical distribution criterion allowed for good coverage. The refusal rate was very low but many households approached had no one at home. Finally, the sample’s higher proportion of women willing to participate may be a sign of participation bias. To further explore if this relationship is causal, future studies should employ a cohort design to establish temporality and develop more sophisticated measures of environmental risk perception related to governmental OSS. In addition, a more rigorous record of OSS interventions during the season will an opportunity to determine the impact of exposure to OSS in addition to perception of its effectiveness on the use of other prevention and control strategies.

### Conclusions

Lower frequencies of elimination of potential mosquito breeding sites, higher exposure to the vector, and higher entomological risk from the residents citing OSS as a way to prevent dengue may support the argument of a “false sense of security”. Further investigations which follow community members over time are needed to establish causality. However, these results can be used to profile residents who may benefit from additional messaging.

It is important to reinforce the message, especially among residents in favor of OSS, that dengue prevention and control is not the sole responsibility of the local government [24]. Practices that provide only partial or unknown levels of protection must be strongly coupled with an emphasis on integrated vector control strategies that include high levels of community participation. The implications of this work can be extended to the roll-out of the new dengue vaccine which offers only partial protection. Careful follow-up should be made to ensure that there is not a decline in other prevention practices following its implementation, especially considering it is only partially effective, a concern also noted by others [7,44]. Although our study is focused on the perception of OSS as a dengue prevention strategy, it can also be applied more broadly to Zika and chikungunya recently introduced into Mexico and also transmitted by *Ae*. *aegypti* [4].

## Supporting information

S1 Checklist STROBE checklist. (DOCX)
